# *HLA-DQA1* and *HLA-DQB1* in Celiac disease predisposition: practical implications of the HLA molecular typing

**DOI:** 10.1186/1423-0127-19-88

**Published:** 2012-10-11

**Authors:** Francesca Megiorni, Antonio Pizzuti

**Affiliations:** 1 Department of Experimental Medicine, “Sapienza” University of Rome, Viale Regina Elena, 324, Rome, Italy

**Keywords:** Celiac disease, *HLA-DQA1*, *HLA-DQB1*, HLA typing, Disease risk

## Abstract

Celiac disease (CD) is a multifactorial disorder with an estimated prevalence in Europe and USA of 1:100 and a female:male ratio of approximately 2:1. The disorder has a multifactorial etiology in which the triggering environmental factor, the gluten, and the main genetic factors, Human Leukocyte Antigen *(HLA)-DQA1* and *HLA-DQB1* loci, are well known. About 90-95% of CD patients carry DQ2.5 heterodimers, encoded by DQA1*05 and DQB1*02 alleles both in *cis* or in *trans* configuration, and DQ8 molecules, encoded by DQB1*03:02 generally in combination with DQA1*03 variant. Less frequently, CD occurs in individuals positive for the DQ2.x heterodimers (DQA1≠*05 and DQB1*02) and very rarely in patients negative for these DQ predisposing markers. HLA molecular typing for Celiac disease is, therefore, a genetic test with a negative predictive value. Nevertheless, it is an important tool able to discriminate individuals genetically susceptible to CD, especially in at-risk groups such as first-degree relatives (parents, siblings and offspring) of patients and in presence of autoimmune conditions (type 1 diabetes, thyroiditis, multiple sclerosis) or specific genetic disorders (Down, Turner or Williams syndromes).

## Review

### Introduction

Celiac disease (CD, MIM 212750) is a chronic gluten-intolerance that occurs in genetically predisposed individuals [1-4]. In sensitive individuals, the ingestion of gluten determinates chronic inflammation of the small intestinal mucosa that results in villous atrophy, crypt hyperplasia and lymphocyte infiltration, leading to nutrient malabsorption. A wide spectrum of clinical phenotypes is present, ranging from classical gastrointestinal manifestations to only atypical signs (Table 1) [5,6]. CD symptoms are frequently represented by the “iceberg model” in which the tip corresponds to patients with classic malabsorption, while the more atypical presentations are included in the invisible and larger portion below the waterline [7,8]. As a general rule, Celiac disease diagnosis can be established by serological tests, searching for anti-tissue transglutaminase (anti-TG2) and anti-endomysium (EMA) auto-antibodies, but confirmation of the intestinal damage relies on the small bowel biopsy and histological analysis, mainly in Europe [9]. A life-long gluten-free diet is the only available and effective therapy, which leads to normalization of histological and serological parameters and to complete remission of all clinical signs [10,11]. Untreated Celiac disease can lead to several long-term complications, such as malnutrition, autoimmune liver disease, peripheral neuropathy and intestinal malignancies [12]. The prevalence of CD is estimated at about 1:100 in Caucasian population but many cases remain undiagnosed, especially among adult individuals, because of the wide variability of symptoms [1,8]. As for other autoimmune diseases, CD occurs more often in female than in male subjects with a gender ratio of about 2:1 [1,13,14]. Furthermore, gluten intolerance is more frequent in at-risk groups, such as first-degree relatives of patients as well as individuals with specific genetic syndromes (Down, Turner, Williams) or autoimmune diseases (mainly type 1 diabetes, thyroiditis and multiple sclerosis) [15-17]. 

**Table 1:** Clinical manifestations of Celiac disease

***Typical symptoms***	***Atypical symptoms***
Abdominal cramping	Dermatitis herpetiformis
Stomach distention	Osteopenia, osteoporosis
Diarrhea	Iron-deficiency anemia
Flatulence	Bone or joint pain
Malabsorption	Abnormal liver function
Weight loss	Hypertransaminasemia
Nausea, vomiting	Dental anomalies
Steatorrhea	Infertility, miscarriage
	Short stature, delayed puberty
	Mouth ulcers
	Fatigue, weakness
	Patchy hair loss
	Ataxia, seizures
	Depression
	Attention-deficit hyperactivity disorder

CD has a multifactorial inheritance, so it does not depend on specific mutations of a single gene but it is caused by a combination of environmental factors and variations in multiple genes [18,19]. Indeed, viral infections altering intestinal permeability, gut microbiota, breast-feed and timing of gluten introduction in infant diet have been related to CD development [20-22]. Familial aggregation (10-12%) and higher concordance rates of CD in monozygotic than in dizygotic twins (83-86% vs. 11%) have been confirmed, indicating that a strong genetic contribution is involved in the disease occurrence [23,24]. Furthermore, the association with other autoimmune conditions in the same individual or in different members of the same family suggests the existence of common predisposing genes to autoimmunity [25].

Human Leukocyte Antigen (HLA) system is the major CD-predisposing genetic factor and the HUGO Gene Nomenclature Committee ( http://www.genenames.org/) has indicated *HLA-DQA1* and *HLA-DQB1* class II genes as *CELIAC1*. Nevertheless, HLA region alone accounts for approximately 40% of the disease heritability [1,11,18] meaning that other genes are involved in CD susceptibility. Many candidate loci have been studied but only three chromosomal regions, 5q31-q33 (*CELIAC2*), 2q33 (*CELIAC3*) and 19p13.1 (*CELIAC4*), have been officially recognized as genetic predisposing factors. In recent years, genome-wide association studies (GWAS) have identified many non-HLA genes associated with an increased risk of CD, such as those coding for cytokines, chemokines and their receptors, cell adhesion molecules, T- and B-cell activators [26-30]. Anyway, non-HLA genetic contribution to Celiac disease is weak (about 15%) and these polymorphisms are not considered in the calculation of CD genetic risk [8,31]. To date, only HLA molecular typing has a role in the disease clinical management.

### HLA genes and CD

HLA class I and class II genes map on the short arm of the chromosome 6 (6p21.3) and code for cell surface glycoproteins important in the antigen presentation and self-recognition by immune cells [32,33]. HLA class I heterodimers are constituted by an alpha-heavy chain, encoded by *HLA-A*, *B* or *C* loci, and by a small beta2-microglobulin molecule whose gene maps on chromosome 15. HLA class II heterodimers (alpha and beta chains) are specified by genes in the HLA-D region that comprehends HLA-DP (*DPA1* and *DPB1*), DQ (*DQA1* and *DQB1*) or DR (*DRB1* and *DRA*) genes. Overall, the two classes are very similar and show a pocket resembling the immunoglobulin Fab region. HLA molecules are able to bind antigenic peptides and to present them to T lymphocytes. In particular, HLA class I molecules/endogenous antigens are specifically recognized by CD8+ T cells, which activate a cytotoxic response, while HLA class II heterodimers/exogenous antigens are bound by CD4+ T cells that, in turn, trigger a humoral response. HLA class I and II genes are highly polymorphic ( http://www.ebi.ac.uk/imgt/hla/stats.html) and present a strong linkage disequilibrium (LD) in which preferential combinations of alleles are inherited together in the genome more often than expected. The series of alleles at linked loci on a single chromosome is named “haplotype” [34].

**Figure 1:**
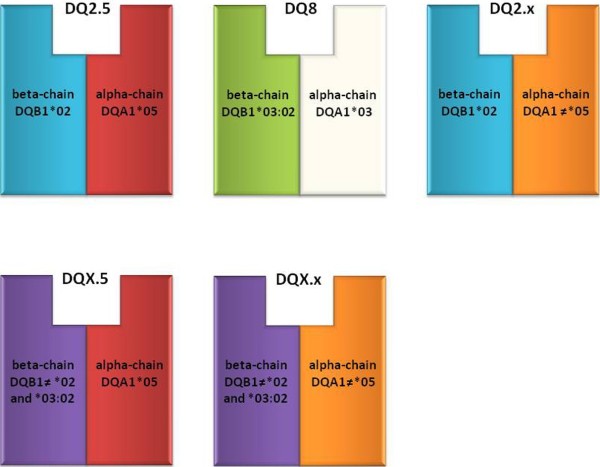
**CD at-risk DQ heterodimers encoded by different combinations of *DQA1*and *DQB1*alleles.**

Approximately 90% of celiac subjects present HLA-DQ2 heterodimers, hereafter called DQ2.5, encoded by DQA1*05 and DQB1*02 alleles, which may be inherited together on the same chromosome (*cis* configuration) or separately on the two homologous chromosomes (*trans* configuration). Generally DQA1*05 and DQB1*02 are present in *cis* on DR3 haplotype (DRB1*03:01-DQA1*05:01-DQB1*02:01) or in *trans* on DR5/DR7 haplotypes (DRB1*11/12-DQA1*05:05-DQB1*03:01; DRB1*07-DQA1*02:01-DQB1*02:02). Numerous studies have also confirmed that DQB1*02 homozygosity is usually associated with increased risk and more aggressive forms of Celiac disease. Almost all DQ2.5-negative patients (5-10%) carry DQ8 heterodimers encoded by DQB1*03:02 allele, generally in combination with DQA1*03 variant in *cis* position on DR4 haplotype (DRB1*04-DQA1*03:01-DQB1*03:02); the majority of DQ2.5/DQ8-negative celiacs (about 5%) presents DQ2.x molecules, encoded by the DQB1*02 at-risk allele in absence of the DQA1*05 variant [1,14,35]. Very rarely, CD patients carry different DQ molecules. Here, I indicate as DQX.5 the heterodimers coded by DQA1*05 allele in absence of DQB1*02 or *03:02 variants while DQX.x molecules are formed by a *DQA1*≠*05 alpha chain and a *DQB1*≠*02 or ≠*03:02 beta chain [14,35]. Interestingly, gender-dependent HLA associations are evident since female patients often carry DQ2.5 and/or DQ8 molecules while DQ2.5/DQ8-negative celiacs are frequently male [14]. Figure 1 summarizes the different DQ glycoproteins with regard to the CD-predisposing alleles. 

The primary HLA-DQ association in CD was initially clarified by experiments on CD4+ T lymphocytes isolated from intestinal biopsies of patients that were able to recognize gluten peptides presented by DQ2.5/DQ8-positive antigen presenting cells [36-39]. In particular, DQ2.5/DQ8 heterodimers display a high affinity for negatively charged amino-acids derived from TG2-mediated deamidation (i.e. conversion of glutamine in glutamic acid amino-acid) [40]. Important interactions occur in a central region of nine amino-acid residues whose lateral chains are anchored to the positions P1, P4, P6, P7 and P9 into DQ-binding pockets. In particular, DQ2.5 heterodimers bind peptides with negatively charged side chains at P4, P6 and P7 positions while DQ8 molecules show a preferential binding for negatively charged residues at P1 and P9 [4]. Gluten-specific T-cell response in patients carrying other DQ molecules (not DQ2.5 or DQ8) has been recently investigated in order to better understand the role played by specific DQ heterodimers in CD pathogenesis [39,41-43]. HLA-DQ-mediated presentation of gluten peptides to CD4+ T lymphocytes is an essential step that stimulates both immune as well as humoral responses. Indeed, cytokine secretion (mainly TNF-α and IFN-γ) induces intestinal fibroblasts to secrete matrix degrading metalloproteinases (MMP-1 and MMP-3) that lead to villous atrophy and crypt hyperplasia. B cell maturation results in the production of auto-antibodies directed against TG2 and TG2-gluten complexes [1,4,10].

### HLA molecular typing in the CD clinical management

So far, only *HLA-DQA1* and *HLA-DQB1* loci have an application in the clinical practice of Celiac disease [44]. HLA test has not a diagnostic significance but is mainly considered for its negative predictive value since CD is highly unlikely when DQ predisposing alleles are absent, while a positive result only means a genetic predisposition for celiac autoimmunity. Indeed, about 30% of the general population carrying DQ2.5/DQ8 molecules do not have the disease and only 3% will develop gluten intolerance. Thus, future investigations should be addressed to identify new markers, which can improve prediction of the disease in people that carry the same HLA at-risk alleles.

Several molecular methods are used for HLA typing (Sequence Specific Primers-PCR, Reverse Dot Blot analysis and Real Time PCR) and, recently, different commercial kits have been developed to specifically genotype CD-associated DQA1/DQB1 alleles (DQA1*05, DQB1*02 and DQB1*03:02) for easier and more rapid tests [45,46]. Some kits also determine the DQB1*02 copy number. Furthermore, even if CD risk is conferred only by *DQA1* and *DQB1* alleles, many molecular kits test particular *DRB1* alleles in LD with the *DQA1* and *DQB1* CD-predisposing variants [45,47]. HLA typing allows to define a CD risk gradient associated with each particular HLA-DQ status (*DQA1* and *DQB1* alleles and their combinations) and DQB1*02 homozygosity/heterozygosity [48,49]. Considering a disease prevalence of 1:100, a very high/high CD predisposition is found in DQ2.5 and/or DQ8 positive individuals and, interestingly, in DQ2.x subjects with two DQB1*02 alleles; a low risk is related to DQ2.x with a single dose of the DQB1*02 variant; people in the DQX.5 and DQX.x categories have an extremely low chance of CD onset (Table 2). 

**Table 2:** HLA-DQ status and risk of Celiac disease

***HLA status***	***Disease risk***
DQ2.5 and DQ8	Very high
DQ2.5 (with a double dose of DQB1*02)	Very high
DQ8	High
DQ2.5 (with a single dose of DQB1*02)	High
DQ2.x (with a double dose of DQB1*02)	High
DQ2.x (with a single dose of DQB1*02)	Low
DQX.5	Extremely low
DQX.x	Extremely low

x = DQA1 alleles different from *05.

X = DQB1 alleles different from *02 and *03:02.

Disease risks for each HLA genotype are based on published data by Megiorni et al. [49].

**Figure 2:**
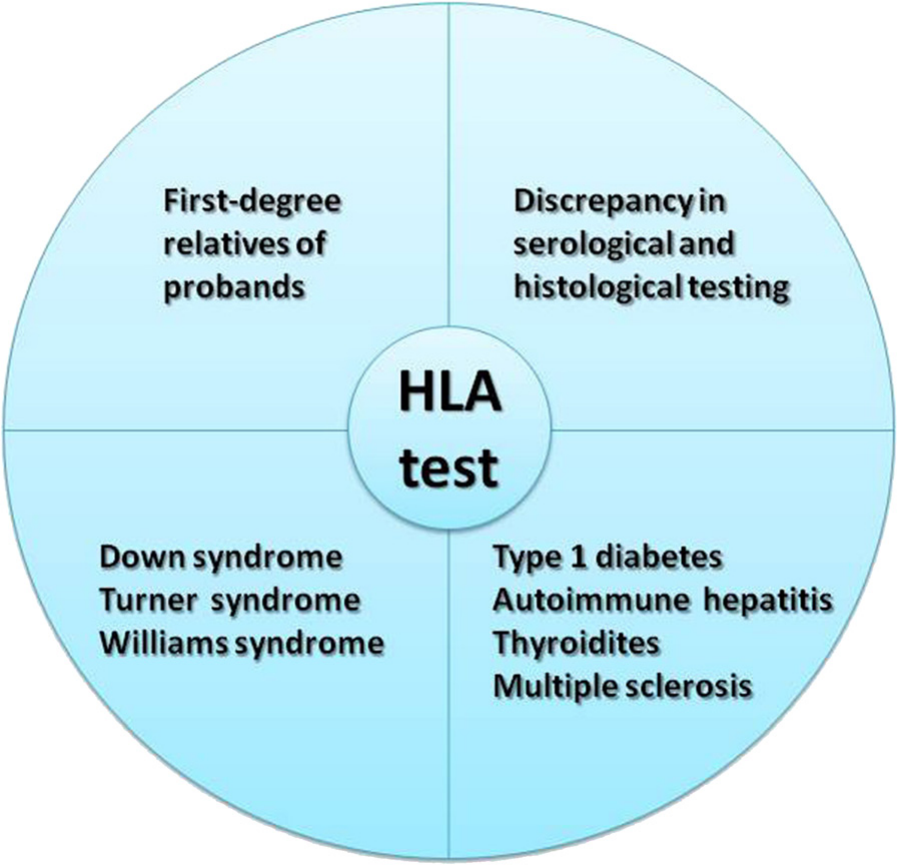
HLA test in at-risk categories.

HLA typing is routinely requested by clinicians to provide additional support in dubious CD cases, i.e. uncertain or discrepant serology and/or biopsy, and in at-risk categories [50,51] (Figure 2). HLA is a useful test in screening first-degree relatives due to the higher prevalence of CD among relatives of celiac patients [24,49]. Family studies showed that celiac autoimmunity occurs almost exclusively in the presence of high-risk DQ molecules (DQ2.5, DQ8 and DQ2.x with a double dose of DQB1*02). Indeed, 20% of sibs and 6% of parents, positive for the *DQA1/DQB1* predisposing alleles, are affected [49], supporting HLA typing as an efficient tool to discriminate individuals who regularly require clinical and serological controls. In this regard, the *DQA1/DQB1* risk gradient could be considered in genetic counseling of CD families to determine more precise disease recurrence risks and appropriate follow-up visits [48,49]. Conversely, a negative gene test result has often a psychological impact since individuals feel reassured of their very low CD risk. 

## Conclusions

Celiac disease is a common multifactorial disorder in which specific *HLA-DQA1* and *HLA-DQB1* alleles represent the major genetic predisposition. HLA typing, however, does not have an absolute diagnostic value but allows to assess the CD relative risk; a positive test is indicative of genetic susceptibility but does not necessarily mean the disease development. A negative test has a more significant value because gluten intolerance rarely occurs in the absence of specific HLA predisposing alleles. HLA genes are stable markers throughout life, so their typing can discriminate genetically CD-susceptible or not susceptible individuals before any clinical or serological signs. HLA test is increasingly considered as a solid support in the diagnostic algorithm of CD. New ESPGHAN guidelines for the diagnosis of CD have established that duodenal biopsy can be omitted in cases with elevated serum anti-TG2 antibodies (>10x upper limit of normal), positive EMA and at-risk HLA [52].

## Competing interests

Non-financial competing interests.

## Authors' contributions

FM wrote the manuscript; AP critically revised the manuscript. Both authors read and approved the final manuscript.
